# Systematic Analysis and Network Mapping of Disease Associations in Autoimmune Polyglandular Syndrome

**DOI:** 10.1210/clinem/dgae701

**Published:** 2024-10-08

**Authors:** Greta Pham-Dobor, Peter Kaltenecker, Viktoria Temesfoi, Laszlo Bajnok, Orsolya Nemes, Beata Bodis, Emese Mezosi

**Affiliations:** First Department of Medicine, Clinical Centre, Medical School, University of Pecs, Pecs 7624, Hungary; Institute For Translational Medicine, Medical School, University of Pecs, Pecs 7624, Hungary; Szentagothai Research Centre, University of Pecs, Pecs 7624, Hungary; National Laboratory on Human Reproduction, University of Pecs, Pecs 7624, Hungary; Szentagothai Research Centre, University of Pecs, Pecs 7624, Hungary; National Laboratory on Human Reproduction, University of Pecs, Pecs 7624, Hungary; First Department of Medicine, Clinical Centre, Medical School, University of Pecs, Pecs 7624, Hungary; First Department of Medicine, Clinical Centre, Medical School, University of Pecs, Pecs 7624, Hungary; First Department of Medicine, Clinical Centre, Medical School, University of Pecs, Pecs 7624, Hungary; First Department of Medicine, Clinical Centre, Medical School, University of Pecs, Pecs 7624, Hungary; Szentagothai Research Centre, University of Pecs, Pecs 7624, Hungary; National Laboratory on Human Reproduction, University of Pecs, Pecs 7624, Hungary

**Keywords:** autoimmune polyglandular syndrome, multiple autoimmune syndromes, autoimmune thyroid disorders, diabetes, autoimmunities, systemic analysis, network mapping

## Abstract

**Background:**

The purpose of our work was to provide a data-driven perspective to autoimmune polyglandular syndrome (APS), a complex autoimmune disorder, supplementing traditional clinical observations.

**Methods:**

Medical records of 7559 patients were analyzed, and autoimmune origin was proved in 3180 cases of which 380 (12%) had APS. Associations of component disorders were investigated by computational methods to reveal typical patterns of disease development.

**Results:**

Twenty-eight distinct autoimmune disorders were diagnosed forming 113 combinations. The 10 most frequent combinations were responsible for 51.3% of cases. Hashimoto's thyroiditis (HT) and Graves’ disease (GD) were differentiated as the main cornerstones of APS, sharing several comorbidities. HT was the most common manifestation (67.4%), followed by GD (26.8%) and type 1 diabetes mellitus (T1D) (20.8%). APS started significantly earlier in men than in women. Thyroid autoimmunity was frequently linked to gastrointestinal and systemic manifestations, and these patterns of associations substantially differed from that of T1D, Addison’s disease, or coeliac disease when present as first manifestations, suggesting the possibility of a common biological cause.

**Conclusion:**

APS is more frequent than reported. Classifying APS requires a shift of perspective toward disease associations rather than disorder prevalence.

Autoimmune polyglandular syndrome (APS) is a complex and heterogenous medical condition in which endocrine and nonendocrine organ-specific and systemic autoimmune disorders coincide. A diagnosis of APS is established when at least 2 organs or organ systems are impacted by autoimmunity (1, 2). Previously it was stated that patients with APS are still not properly diagnosed, and the presenting diseases are frequently treated separately (3). In this work, we aimed to rethink the definitions of APS based on a systematic analysis of disease associations and to initiate financially feasible screening protocols according to a newly identified pattern of subsequent organ system involvement and disease progression.

APS is traditionally divided into 4 subgroups (4). APS I is a monogenic disorder that develops in childhood as a result of mutations in the autoimmune regulator (AIRE) gene and is a combination of Addison's disease (AD), mucocutaneous candidiasis, and hypoparathyroidism (hypoPT) (4).

APS II is described to consist of component diseases such as AD, autoimmune thyroid disease (AITD), and/or type I diabetes mellitus (T1D) (4). A patient with AD has at least a 50% lifetime risk for the development of additional autoimmune disorders (5). AITDs include Hashimoto's thyroiditis (HT) and Graves’ disease (GD) (6). In APS III, AITD can be associated with any other autoimmune conditions except AD (4, 7-9).

Patients who cannot be classified into any of the aforementioned 3 categories fall into APS IV (4, 7-9). According to Gatta et al, T1D is a key element for the diagnosis of APS IV (10). Additionally, the literature offers a few other classifications that are distinct in their definitions. According to Frommer and Kahaly, there are juvenile and adult versions of APS, combining groups II to IV of the traditional classification. Most variations have been described for APS groups II and III (1, 11).

APS III group can be further divided into subgroups. Betterle and colleagues distinguished 4 subtypes based on the associated diseases. Subgroups that can be distinguished on this basis are (1) AITD and autoimmune endocrine diseases; (2) AITD and autoimmune diseases of the gastrointestinal tract, hepato-biliary system, or exocrine pancreas; (3) AITD and autoimmune diseases of the skin, the nervous system, and/or the hematologic system; and (4) AITD and autoimmune rheumatic or autoimmune cardiac diseases, antiphospholipid syndrome, or vasculitis (12, 13). Additionally, this working group refers to APS III as multiple autoimmune syndromes III (12). The rationale behind this nomenclature is that APS III includes endocrine and nonendocrine autoimmune manifestations as well, making it inappropriate to refer to “polyendocrine autoimmunity” in its name (12). Women are more frequently affected by APS than men, as is typical for autoimmune disorders. The syndrome has a peak incidence in the third or fourth decade of life, although the onset of the individual diseases may vary (2, 9, 14-16).

APS is characterized by lymphocytic infiltration of the affected tissues, autoantibodies against endocrine and nonendocrine organs, and disturbances of the humoral and cellular immune response (2, 8, 17-20). In APS II and III, involvement of human leukocyte antigen (HLA), CTLA-4, and PTPN-22 genes has also been observed (15, 19, 21, 22). Both genetic and environmental factors may contribute to the loss of immune self-tolerance by disrupting the communication between antigen-presenting cells and T-cells (14, 15, 23). Patients with APS have higher concentrations of autoantibodies against afflicted organs compared to individuals with single autoimmune diseases (24). Coeliac disease (CeD) is known to be closely associated with T1D due to the fact that these conditions share the same HLA susceptibility alleles, specifically the DR3/DQ2 and DR4/DQ8 molecules (10, 25). There are some other non-HLA genes such as V-set domain-containing T-cell activation inhibitor 1 and receptor IIb, which might play a role in the development of autoimmune disorders and thus APS (15). Polymorphisms within the BTB domain and CNC homolog 2 locus were found to be associated with vitiligo (Vit), rheumatoid arthritis (RA), and systemic lupus erythematosus (SLE). Additionally, BTB domain and CNC homolog 2 variants were also linked to AD and AITD (26). Genetic risk for T1D overlaps with AITD, Crohn’s disease (CD), and AD (27). Beyond the endocrine autoimmune disorders that are obligatory for the diagnosis, both APS II and III can be associated with a wide variety of other nonendocrine autoimmune conditions (9). Autoimmune disorders of the endocrine glands such as AITDs, T1D, AD, premature ovarian failure (POF), hypoPT, or lymphocytic hypophysitis may be combined with nonendocrine organ-specific autoimmune disorders including Vit, alopecia, inflammatory bowel diseases, CeD, autoimmune hepatitis, autoimmune hemolytic anemia, autoimmune gastritis (AIG), multiple sclerosis, or myasthenia gravis. Furthermore, systemic autoimmune disorders such as RA, SLE, Sjögren's syndrome (SS), psoriasis, systemic sclerosis, and polymyositis may also be present as comorbidities (3).

To achieve a thorough and clear understanding of this polyautoimmune syndrome, along with the ability to anticipate future involvement of organ systems or organs, it is essential to prioritize the ongoing monitoring and cooperative care of patients. The purpose of our work is to provide a data-driven perspective to the field, in addition to traditional clinical observations and patient classification strategies, in order to enhance proper diagnosis and predictive possibilities.

## Methods

###  

#### Sex as a biological variable

In this study, we examined both women and men patients with APS. There was no biased selection toward either sexes; indicated ratios represent the real occurrence of women and men in the cohort investigated.

#### Patient selection and inclusion criteria

Our study was conducted at the Division of Endocrinology and Metabolism, First Department of Internal Medicine, Clinical Centre of University of Pecs, Hungary. Data was retrieved from the MedSol database of patients with the following International Statistical Classification of Diseases and Related Health Problems codes since March 2007: E3100—APS, E2711—AD, E2710—adrenal failure, E1080, E1090—diabetes mellitus, E0630—autoimmune thyroiditis, E0500—hyperthyroidism. Charts and medical records of the outpatients were analyzed to prove the presence of at least 2 autoimmune diseases. Data from those who met the criteria were sorted into an Excel spreadsheet (Microsoft Excel 2021, v. 2405) in which the following were indicated: age at the diagnosis of the first manifestation, sex, corresponding APS classification, first and second manifestations, and elapsed time between the diagnosis of these 2. Where it occurred, third and fourth manifestations were recorded as well. In addition, the Supplementary Table lists all autoimmune manifestations appearing in the cohort (28). In some cases, the APS was diagnosed at the first visit at our clinic, while in other cases, the appearance of a new autoimmune disease was detected during the follow-up. During the building of this database, all the patients’ records were studied retrospectively to obtain the most precise information possible. No specific diagnostic flowchart was followed; the disorders were diagnosed based on clinical suspicion with the routinely available diagnostic tools. All autoantibody assays were requested based on clinical suspicion for an autoimmune disorder. Patients positive to anti-thyroglobulin and anti-thyroid peroxidase autoantibodies were classified having autoimmune thyroiditis. Most of these patients had hypothyroidism. The final diagnosis of systemic autoimmune disorders was completed after longer follow-up to avoid the misclassification. Other organ-specific autoimmune disorders were not diagnosed without functional or clinical abnormalities.

#### Data handling and statistics

Data handling and manipulation were done both in Python (v. 3.11.4) and R (v. 4.3.1). For this purpose, we used the numpy (v. 1.24.4) and pandas (v. 2.0.3) packages in Python, whereas the R base package (v. 4.3.1) and the Tidyverse library (v. 2.0.0) were utilized in R. The statistical analysis was performed in R. In each case, Shapiro–Wilk tests were used to examine the normality of the data. For comparing only 2 groups/conditions, we used unpaired two-sample Student's *t*-tests if the data were normally distributed within each group; if not, nonparametric unpaired two-sample Wilcoxon rank sum tests (Mann–Whitney) were applied. For multiple comparisons, since at least 1 of the compared groups/conditions always showed a nonnormal distribution, we utilized the Kruskal–Wallis rank sum test first. In case of significancy, it was followed by post hoc Dunn's multiple comparisons tests (Bonferroni method). Similarly, we applied the same Kruskal–Wallis and Dunn tests to investigate the difference between several groups with categorical variables. During the complete analysis, the confidence interval was defined as 95% in each test. Although the function applied to do Dunn's multiple comparisons tests is part of the FSA library (v. 0.9.5), all functions for the other tests are from the R stats package (v. 4.3.1). Besides these, basic descriptive statistics such as mean, median, SD, and minimum and maximum values were calculated in Python as well with numpy and pandas.

#### Network analysis

The graph for network analysis was created with networkx (v. 3.1) in Python. Nodes (diseases) and edges (co-occurrence of diseases) with their weights (number of co-occurrences) were manually added based on the collected data from the total cohort. We produced 3 visual representations of this graph in which the position of nodes is defined by different algorithms. The spring layout uses a force-directed algorithm (Fruchterman–Reingold) to draw the graph. The Kamada–Kawai layout utilizes its own path-length cost function to create an alternative force-directed layout, whereas the circular layout positions nodes on a circle (29). In each case, the degree of a node (the number of connections a node has) is indicated by its size. Edge weights are represented by the length of the edge (spring layout) or by the color darkness of the edge (circular layout). To guarantee the reproducibility of these visualizations, we used the random module of Python and the random function of numpy to set the same seed (not applicable in case of circular mapping). Communities in the graph were detected by Louvain community detection algorithm implemented into networkx (30). All edge weights were considered during detection, and resolution was set to 1.1 in order to find relatively large clusters. The association of autoimmune diseases with HT and/or GD was also examined in networkx: the neighbors of each node are easily accessible by the so-called adjacency object of the graph.

#### Data visualization

To visualize the data, diagrams were made with matplotlib (v. 3.7.2) and seaborn (v. 0.13.2) in Python and with ggplot2 (v. 3.4.4) and ggthemes (v. 5.0.0) in R. End points of the violin plots are cut at the minimum and maximum values of the data range. Box plots within the violin plots represent data distribution as follows: quartiles of the dataset are indicated by the box and median is labeled, while the rest of the distribution is shown by the whiskers except for outliers. Outliers were identified as data points that are beyond the first and third quartiles with 1.5 times the interquartile range. Sankey diagrams were constructed using the online tool SankeyMatic created by Steve Bogart (https://sankeymatic.com). These diagrams show APS development along the successive manifestations: the initial disease of APS is on the left from where newly emerging associated illnesses are drawn in a branched structure. The width of the horizontal bands is proportional to the frequency of connections in comparison to the others. The percentages displayed indicate the ratio of major second manifestations that arise from the same disease in relation to each other. In addition, we used a dimensionality reduction method for visualization purposes as well, uniform manifold approximation and projections (UMAPs) were made with the umap-learn package (v. 0.5.3), and with all its dependencies, in Python. The resulting projections were created based on the co-occurrence of APS diseases. In these mappings, each data point represents a single patient, and the position of the point is defined by the disease combination that belongs to the actual patient. The distance between points/patients indicates the degree of similarity; therefore patients with more similar disease combinations will appear closer to each other in the plot. To visualize the co-occurrence of diseases in a traditional way, a coordinate system with 28 axes, as many as the number of illnesses, should be drawn and observed, which encounters serious difficulties. The UMAP algorithm solves this problem and translates this information by mapping it to a 2-dimensional space. The same settings were applied for all UMAPs: the distance metric was Manhattan distance; the number of neighbors was 15 for estimating the manifold structure of the data and the minimum distance between points in the low dimensional representation was set to 0.2. For accurate reproducibility, the same random seed was provided for all UMAPs. Final adjustments of the figures were made in Procreate (Savage Interactive Pty Ltd, v. 5.3.7) where necessary.

#### Study approval

The study was conducted according to the guidelines of the Declaration of Helsinki and approved by the Regional and Institutional Research Ethics Committee of the Medical School University of Pecs, Hungary (protocol code 7852-PTE 2019).

## Results

### 67.4% of Individuals Diagnosed With APS Were Found to Have Hashimoto's Thyroiditis as 1 of the Manifestations

A sampling of 380 patients was analyzed from a database of 7559 total cases recorded at the Division of Endocrinology and Metabolism, First Department of Internal Medicine, Clinical Centre of University of Pecs, Hungary, containing all patients who appeared in the system between 2007 and 2018 with a diagnosis of thyroid dysfunction, AITD, AD, T1D, POF, lymphocytic hypophysitis, or hypoPT. The patients were followed up until the December 31, 2023; 3180 patients (49.06%) were diagnosed with autoimmune endocrine disorders. Twelve percent of patients showed more than 1 autoimmune manifestation with at least 1 that affects the endocrine organ system and thus were classified into the APS categories.

**Figure 1.**
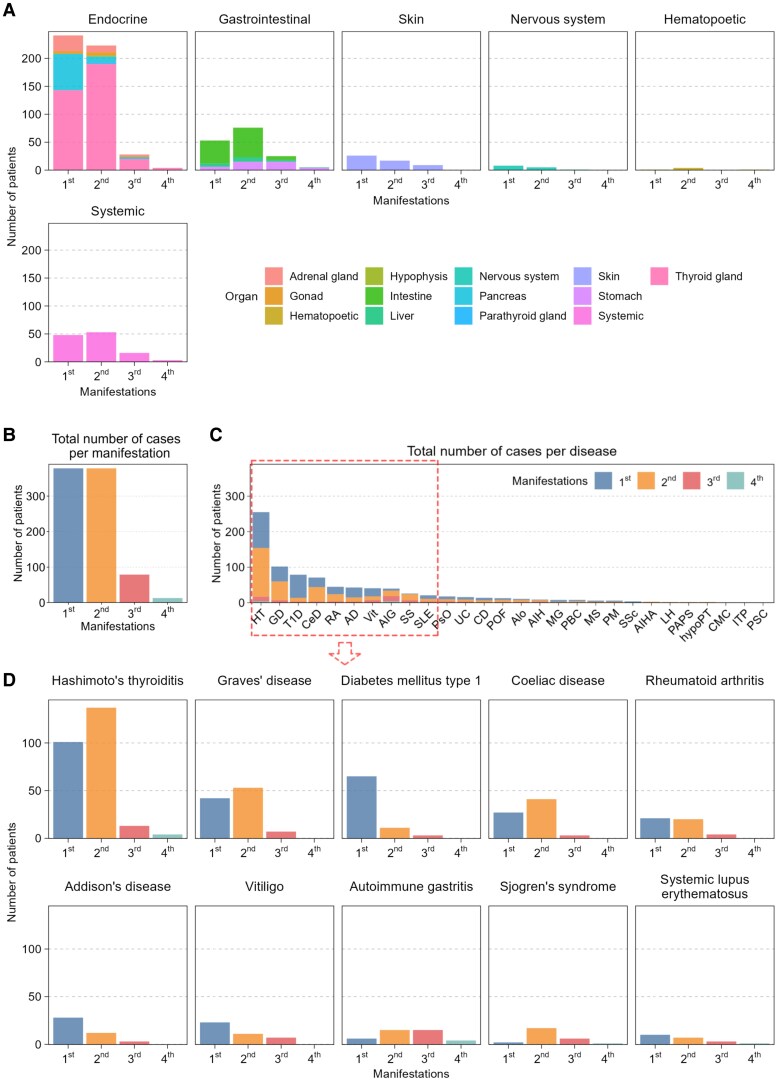
Exploratory statistics of the affected organ systems and incidence of component diseases in the manifestations of APS. (A) Involvement of organ systems in the APS population by manifestations. (B) Absolute number of cases per manifestation. (C) Number of cases per disease in the manifestations. (D) Occurrence of the 10 most prevalent diseases across the 4 manifestations. Abbreviation: APS, autoimmune polyglandular syndrome.

Due to the diagnostic criteria of APS, most autoimmune illnesses in the study population were predominantly associated with the endocrine system, whereas the gastrointestinal system was the second most frequently affected. Systemic conditions, such as RA, SS, and SLE were the third most common component diseases in our population. Conditions affecting the skin were also present in significant numbers (Fig. 1A).

It is important to mention that according to the characteristics of the study we worked on a snapshot in which we have not witnessed all the possible manifestations in all the cases yet, but we were able to identify the presence of at least the initial 2 manifestations, which is a necessary requirement for diagnosing APS. Consequently, 2 manifestations are the prevailing scenarios in our population, whereas 17.4% of the cases presented 3 and 3.7% 4 manifestations (Fig. 1B).

The 10 most frequent disorders were HT, GD, T1D, CeD, RA, AD, AIG, Vit, SS, and SLE (Fig. 1C). The most prevalent condition in terms of both primary and secondary manifestations was HT. A comparable trend could be observed in the occurrence of GD in the manifestations, albeit with a reduced incidence rate. T1D was considerably more likely to manifest as the initial symptom of APS rather than as a superimposed or additional condition. The most common third manifestation was AIG, preceding other frequently observed diseases, including HT, GD, and T1D. AIG and SS were less probably the initial symptoms of APS (Fig. 1D).

### Two Peaks Around the Ages of 20 and 40 Are Detected in Both Sexes When the Manifestations of APS Appear With a Consistent Age Difference of 7 to 8 Years Between Men and Women

**Figure 2.**
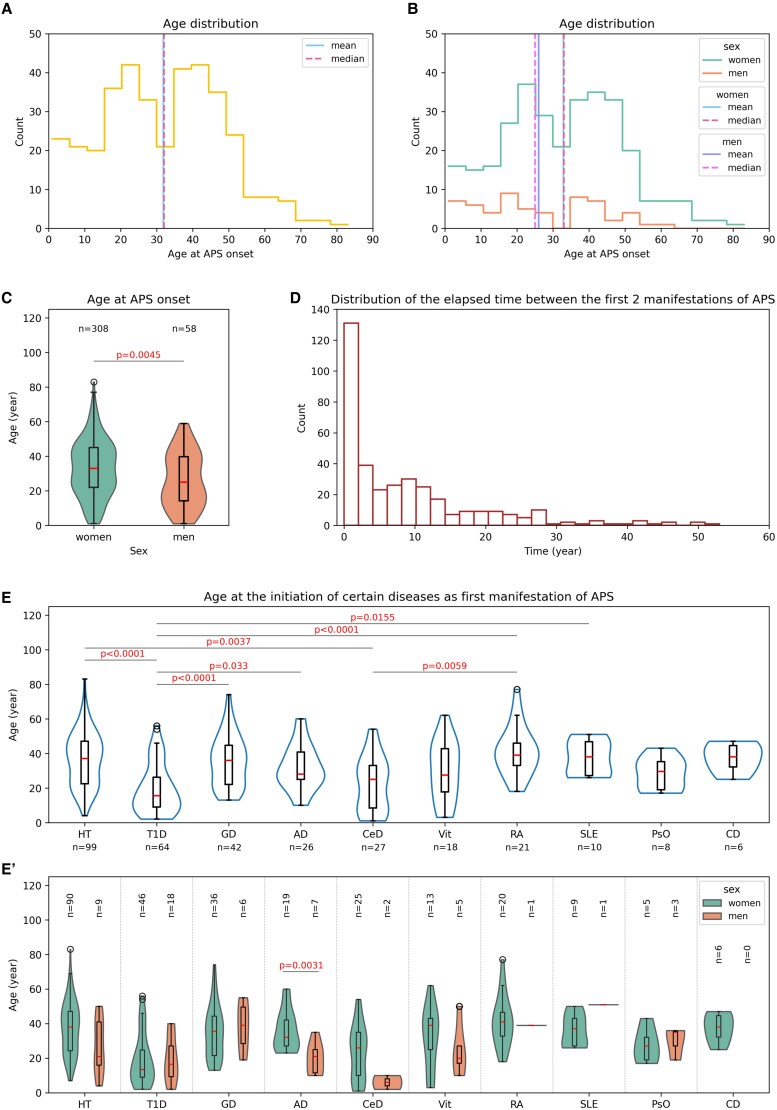
Detailed statistics on age-related information obtained from the APS population. (A) Age distribution of the entire population, including both mean and median values. (B) Age distribution of the APS population divided by sexes, including mean and median values. (C) Age distribution at disease onset in men and women. (D) Distribution of the elapsed time between the first 2 manifestations of APS. (E) Age at the initiation of the 10 most frequent disorders as first manifestation of APS. (E') Age at the initiation of the 10 most frequent disorders as first manifestation of APS by sexes. Abbreviation: APS, autoimmune polyglandular syndrome.

Considering the age of the studied population at the initiation of APS, all the age groups were represented, ranging from the youngest patients from 1 year old to the maximum age of 83 at the onset of the disease. The average age at which APS started was 31.8 years for the entire study population, with a median of 32 (Fig. 2A). Women had a higher prevalence, accounting for 84.2% of the total, in contrast to the 15.8% represented by men as shown in Fig. 2C. The average age for women was 32.9 years, with a median value of 33, while the mean age for men was 26 years, with a median of 25. The data show that the disease tends to manifest in men around 7 to 8 years earlier on average compared to women (Fig. 2B and 2C). In both sexes, 2 peaks could be observed around the ages of 20 and 40 showing an elevated number of disease onsets, aligning with the aforementioned age difference between men and women (Fig. 2A and 2B).

As mentioned earlier, the most prevalent scenarios in our study were the 2 manifestations, which are commonly identified together when patients seek medical attention due to an accumulation of clinical symptoms; therefore, in 67 cases, the elapsed time between the first and second manifestations was registered as 0 years. Furthermore, we may come across a wide range of scenarios, in which the timespan between the first 2 manifestations might vary from 1 to 53 years (Fig. 2D).

### T1D and CeD Manifest the Earliest Among the APS Component Diseases When Presenting as the Initial Symptom

Notable variations could be observed in the age at which autoimmune conditions first appeared. The 2 earliest developing diseases among the most common first manifestations were T1D and CeD with an age distribution where a large number of incidences could be seen under the age of 20. T1D appeared significantly earlier than most of the other frequent first manifestations, such as HT, GD, AD, RA, and SLE. CeD came second and Vit third in this kind of comparison. There was no apparent statistically significant difference observed among HT, GD, AD, RA, and SLE (Fig. 2E). By breaking down this data based on sex, it became clear that the onset of AD typically occurred at an earlier age in men when presented as the initial symptom (Fig. 2E’).

### Network Analysis of Disease Associations in APS

**Figure 3.**
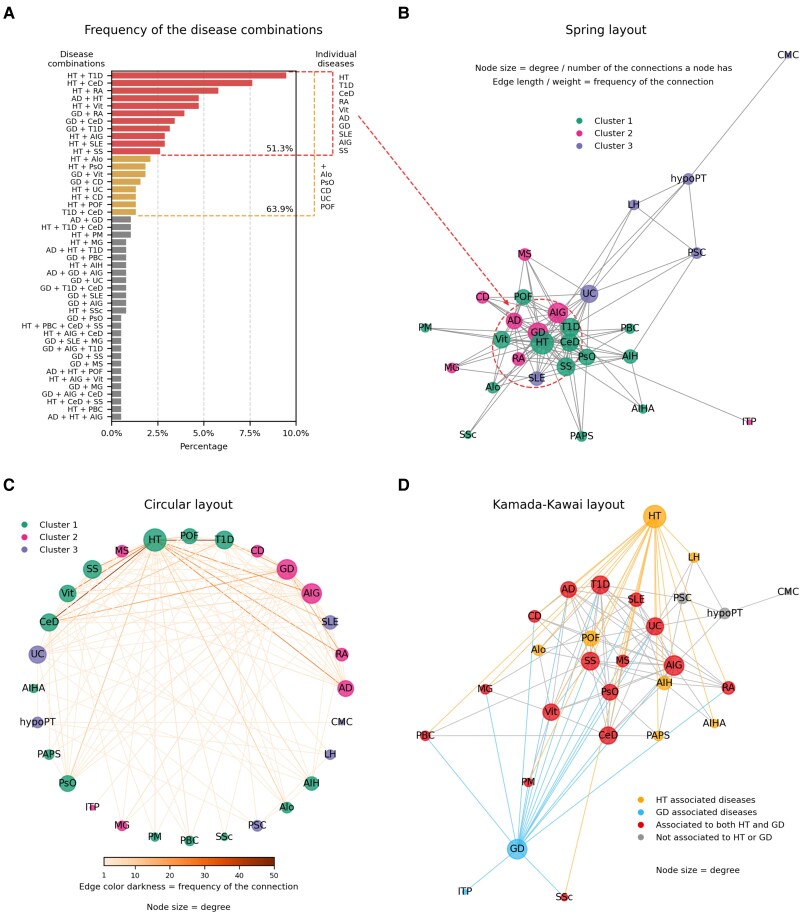
Analysis of disease associations in APS using frequency and network visualization. (A) Frequency of disease combinations showing the associations that occurred more than once in the population regardless of the order of disease appearance. Most prevalent combinations, which appeared at least 5 (yellow) or 10 times (red), are highlighted in color. (B) Network mapping of the APS component diseases using the Spring layout. The 10 most frequent diseases are labeled with a dashed circle. Node coloring indicates the categorization of diseases according to the Louvain algorithm. (C) Network mapping of the APS component diseases in a circular layout. Node coloring indicates the categorization of diseases according to the Louvain algorithm; the darkness of the connecting lines depicts how frequent a combination is in the population. (D) Network of diseases in a Kamada–Kawai representation based on their connections to the 2 thyroid-related disorders. Illnesses exclusively linked with HT are labeled with yellow (6), while illnesses exclusively associated with GD are labeled with blue (0). Red is used to illustrate components related to both HT and GD (15), while diseases not associated directly with HT or GD are represented by the color grey (3). Abbreviations: APS, autoimmune polyglandular syndrome; GD, Graves’ disease; HT, Hashimoto's thyroiditis.

In our patient population, 28 separated autoimmune disorders were diagnosed. A total of 113 combinations of diseases could be detected, 46 of which occurred more than once, as highlighted in Fig. 3A. Combinations that were individual or unique accounted for 17.6%. Numerous combinations were very rare, but those occurred at least 5 times or more, making up over half (63.9%) of the population. Narrowing it further down to those occurring at least 10 times or more, the selection still represented 51.3%. These most frequent combinations consisted of only dual manifestations of the 10 most prevalent diseases in the population, listed previously. Due to the nature of data collection, combinations of 3 or more diseases were less represented (Fig. 3A).

Connections of each individual disease are visualized in Fig. 3B, 3C, and 3D regardless of the chronological order of their appearance. Diseases are depicted as nodes in the network, with the links between them illustrated by the connecting lines (edges). Size of the nodes indicates the degree of adjacency with other diseases: the bigger the node size, the more connections the disease has. Weight was applied to each connection in the network according to the frequency of 2 diseases occurring together. Proximity of the nodes correlates with a higher frequency of co-occurrence of 2 conditions. The core of the representation in Fig. 3B highlights the 10 most prevalent diseases. Figure 3C presents the same information in a circular layout, enhancing comprehension by using color darkness on the lines to indicate the closeness of relationships instead of the length of edges. The darker the line, the closer the relationship between 2 diseases.

An important but not surprising insight gained from the network mapping is that HT was not connected to GD in any case (Fig. 3D). Due to their biologically contrasting nature, none of the patients showed simultaneous occurrence of the 2 disorders. The most prevalent associations were related to HT involving T1D, CeD, RA, Vit, AD, SLE, AIG, or SS in a decreasing order. The second most frequent set of concurrent presence involved GD associated with RA, CeD, or T1D. The clustering algorithm effectively differentiated HT and GD in the network and organized associated disorders so that they are more densely connected within their own community than with nodes in other communities (Fig. 3B and 3C). Still, the related diseases to HT and GD were not completely separated, as is visible in Fig. 3D. The Kamada–Kawai layout initially placed GD and HT on the 2 edges and very well outlined how the network of the 2 diseases is intertwined. HT and GD share many comorbidities: 15 in total. There was only 1 disorder that was associated with GD but not with HT (immune thrombocytopenia), 6 connected to HT but not to GD (alopecia, POF, autoimmune hepatitis, primary antiphospholipid syndrome, autoimmune hemolytic anemia), and 3 that were not in close relation with either of them (psoriasis, hypoparathyroidism, mucocutaneous candidiasis).

### T1D and HT Are the Most Common Component Diseases in the Juvenile Cohort

**Figure 4.**
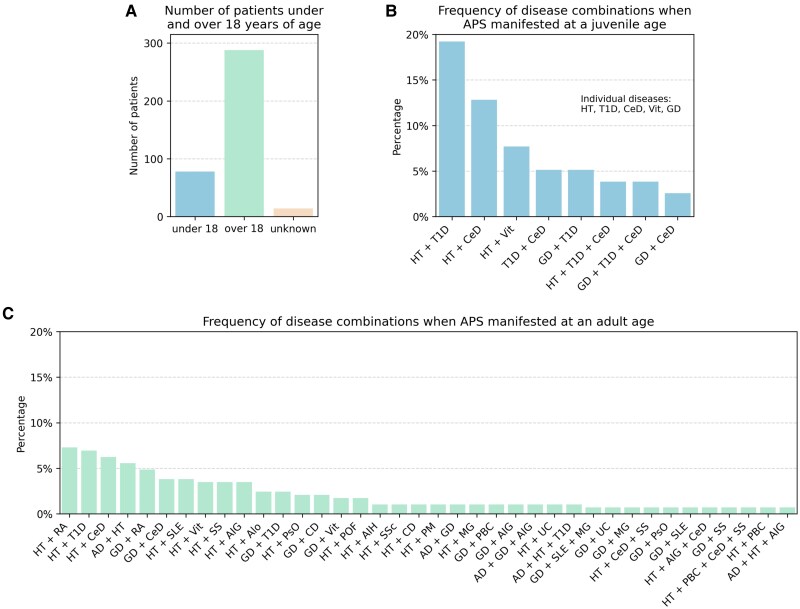
Comparative analysis of the frequency of disease combinations in individuals under and over 18 years of age. (A) Number of patients under and over 18 years. (B) Frequency of disease combinations manifested under 18 years of age regardless of the order of appearance. (C) Frequency of disease combinations manifested over 18 years of age regardless of the order of appearance. Unique combinations are excluded from this analysis; (B) and (C) show only the combinations that appeared more than once.

While the age at which APS first appeared was unclear in 3.7% of cases, the majority of individuals developed the first symptom after the age of 18, with just 20.5% experiencing it before turning 18 (Fig. 4A). We hypothesized that the APS component diseases in childhood might differ in terms of combinations. Regardless of the order of appearance of the component diseases, the most prevalent combination in those who presented the first manifestation of APS in childhood was T1D with HT, which meant 19.2% of the cases. It is noteworthy that T1D and HT both appear in at least 50% of the combinations that occurred more than once, respectively (Fig. 4B). Looking at the adult cohort, the most common scenario was the concurrent presence of HT and RA. However, there were also numerous cases where HT was associated with T1D, CeD, or AD (Fig. 4C).

### The First Manifestation Is Likely to Impact the Development of Subsequent Disorders in APS

**Figure 5.**
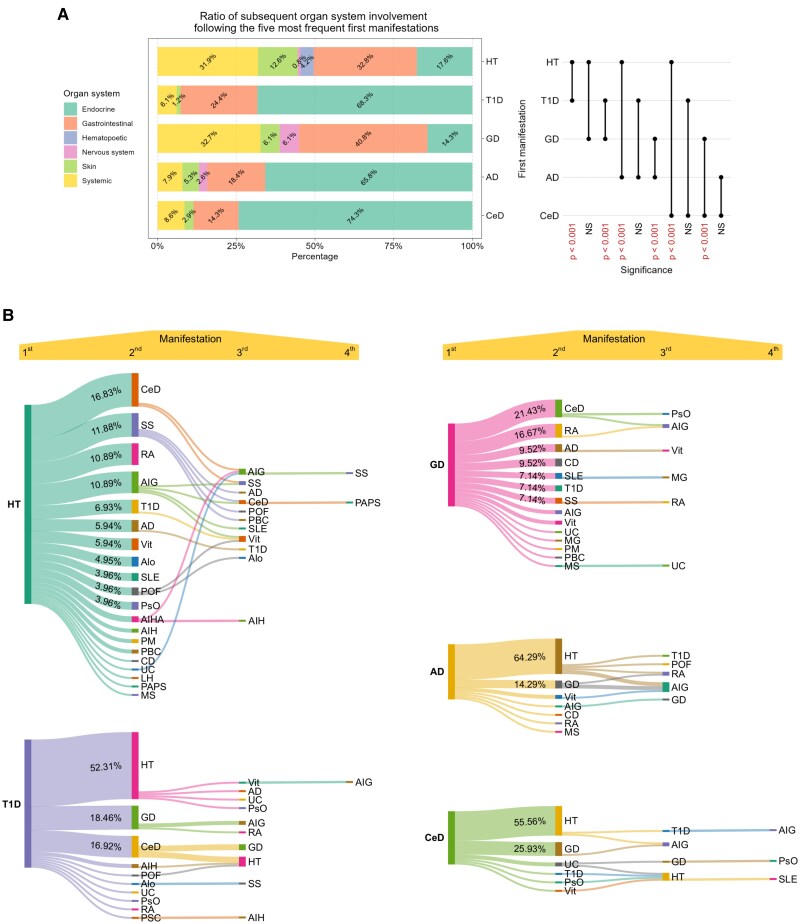
Subsequent organ system involvement and disease progression following the most frequent initial manifestations. (A) Ratio of subsequent organ system involvement following the 5 most frequent first manifestations. Statistical difference is depicted in the lollipop plot. (B) Mapping the progression of APS in a branch representation based on disease development following the 5 most common initial symptoms. Nodes indicate the diseases while horizontal bands display the different routes. Abbreviation: APS, autoimmune polyglandular syndrome.

An interesting pattern becomes apparent when examining the organ systems that were affected following the 5 most observed first manifestations. Diseases affecting the thyroid gland (HT and GD) displayed a unique composition in terms of the later organ system involvement compared to the other 3 investigated first manifestations (T1D, AD, CeD). Patients diagnosed with HT or GD had a high prevalence of gastrointestinal (32.8% and 40.8%, respectively), as well as systemic (31.9% and 32.7%, respectively) diseases in the latter phases. In contrast, following T1D, AD, and CeD, the endocrine system was predominantly involved in the later manifestations (68.3%, 65.8%, and 74.3%, respectively), with less but still relevant gastrointestinal (24.4%, 18.4%, and 14.3%) and systemic (6.1%, 7.9%, and 8.6%) involvement. The observed variance in this pattern exhibited a strong and statistically significant difference (Fig. 5A).

Tracking the course of disease progression as each patient went through APS, significant routes could be observed. Interestingly, the pattern described previously can be seen in this case as well: the composition of subsequent diseases associated with HT or GD differed from that of those related to T1D, AD, or CeD. HT as first manifestation might be followed by a wide variety of diseases, which occurred in approximately similar proportion, with CeD being the most prevalent (16.8%). SS, RA, and AIG were observed in these patients with a frequency exceeding 10% each (Fig. 5B). Similarly to HT, CeD was present in the largest proportion as secondary manifestation (21.4%) in patients with GD, while RA was the second (16.7%), AD the third (9.5%), and CD the fourth (9.5%) most frequently acquired disease. Patients who had T1D as the initial symptom developed HT in 52.1% of the cases, which was remarkably higher than any other conditions that appeared, such as GD (18.5%) or CeD (16.9%) as secondary manifestation (Fig. 5B). A considerable proportion of AD patients also developed HT (64.3%), making it the most common secondary manifestation, followed by GD at 14.3%. When CeD is the primary symptom, similar ratios could be observed, with HT being the most common (55.6%) and GD being the second most frequent (25.9%) secondary manifestation (Fig. 5B). No diseases could be observed affecting the nervous system in cases where T1D or CeD was the initial symptom. Two cases where the first manifestation could not be determined were left out of this analysis. Although in this section we present only the 5 most prevalent diseases, the mapping of subsequent manifestations through APS (Supplementary Table S1) contains this information for all diseases that appeared as first manifestations (28).

### HT and GD Are Probably the Two Cornerstones of APS Among the Component Diseases

**Figure 6.**
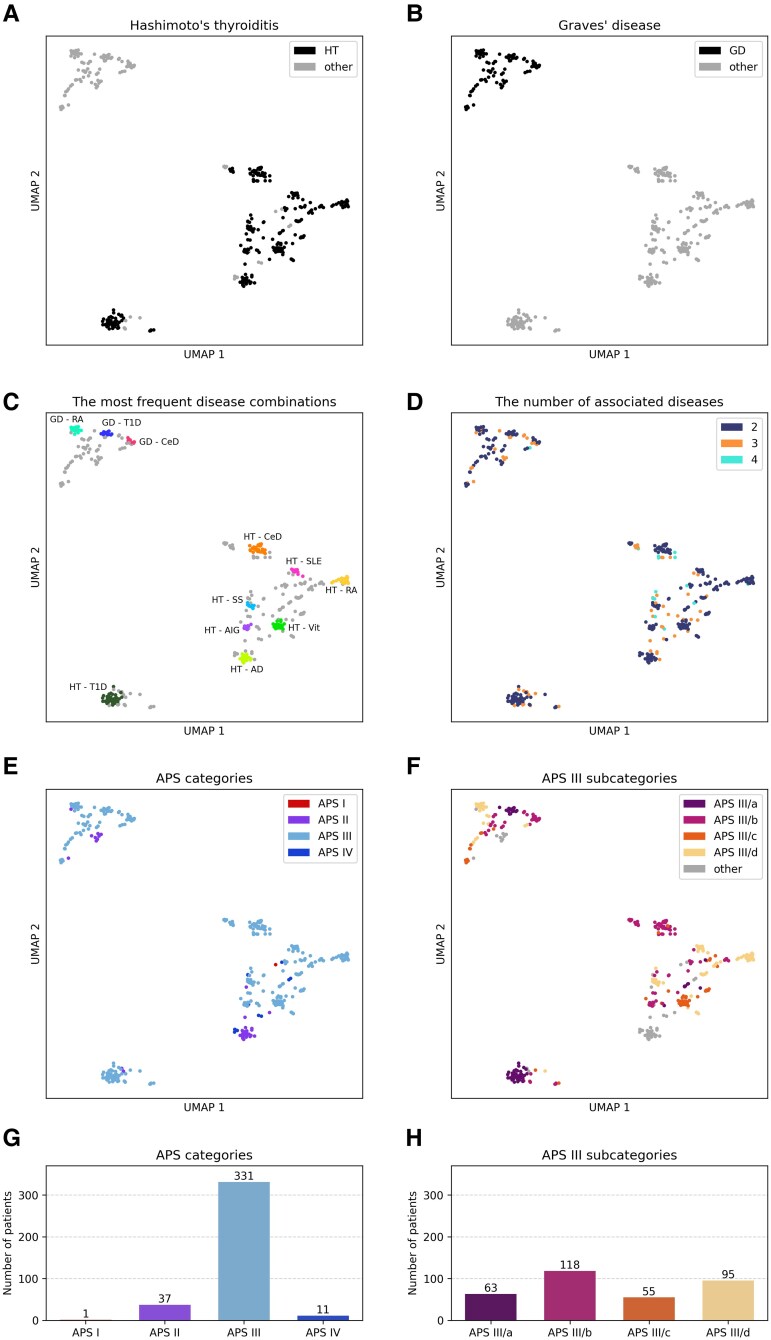
Visualization of the APS cohort in a dimensionally reduced space using the UMAP projection. (A) Localization of the patients with HT as 1 of the manifestations in the UMAP projection. (B) Localization of the patients with GD as 1 of the manifestations in the UMAP projection. (C) Position of the most frequent disease combinations. (D) Depiction of patients with 2, 3, and 4 acquired manifestations. (E) Mapping of APS categories on the cohort. (F) Mapping of APS III subcategories on the cohort. (G) Patients’ distribution in APS groups (H) Patients’ distribution in APS III subgroups. Abbreviations: APS, autoimmune polyglandular syndrome; GD, Graves’ disease; HT. Hashimoto's thyroiditis; UMAP, uniform manifold approximation and projection.

Diseases and disease combinations were visualized in a dimensionally reduced space to facilitate comprehension of the local and global relationship system. Similarly to the result of the network analysis, HT and GD are unequivocally separated on the UMAPs (Fig. 6A and 6B). Interestingly, the group of patients that make up the combination of HT and T1D is depicted even more separated from other combinations involving HT, prompting further speculation regarding the underlying factors (Fig. 6C). Based on the mapping of the number of disease associations, the visibly cohesive clusters that are more distinct from the background mainly consist of combinations formed by 2 diseases. When comparing this with the most frequent associations in our data, we realized that the 11 combinations that we identified as the most common make up these clusters (Fig. 6D). Furthermore, it is also visible that these groups of cases do not completely coincide with the APS categories (Fig. 6E). Although the system of grouping is somewhat similar between the 2 methods, the current clinical classification of APS fails to acknowledge the association between a specified condition and HT or GD. This can be observed, eg, in the APS III/a category where the classification is determined by the presence of T1D. Similarly, the majority of APS III/b consists of patients with CeD, regardless of their association with HT or GD, causing the spatial separation of these APS subgroups in the UMAP visualization (Fig. 6F). However, as shown in our analysis, the patients in the APS III/a, and III/b subgroups clearly tend to divide into 2 directions based on their connection to HT and GD.

The localization of the 10 most frequent individual disorders in the dimensionally reduced space and the sex and age distribution are depicted in Supplementary Fig. S1 (28)

## Discussion

APS is a complex, rare—or thought to be rare—disease. Its prevalence ranges from 1:100 000 to 1: 20 000 according to the literature (1). However, our data suggest that it is much more common, occurring in 12% of the 3180 patients with endocrine autoimmune diseases in our database. Our study is 1 of the most extensive patient communications regarding APS (31, 32). The observed prevalence of this disorder was unexpectedly high considering the previously published international data, which leads to the question of the true occurrence of the disease. However, our clinic is a tertiary center, which may create a selection bias by including more complicated endocrine cases. Also, the study was conducted at a single center. The results solely indicate the present condition of the patients, without any more follow-up information being accessible. Regrettably, owing to the inherent characteristics of the diseases, it proved challenging in numerous instances to ascertain the initial manifestation of each ailment. Consequently, it was not possible to analyze certain data in relation to the first and second manifestations.

However, in this study we introduced an analytical strategy that focused entirely on the associations of the individual diseases and differed from the traditional patient classification methodology and has not yet been used in assessing the development of APS. We have found 28 different autoimmune disorders with 113 combinations, but more than half of the patients belonged to the 10 most common associations. More than two-thirds of the patients had HT, ranking this disease as the most common disorder.

In relation to the terminology, as not only endocrine organs were impacted but gastrointestinal, systemic, and cutaneous autoimmunity also manifested among the top 10 most common conditions, it is worth considering the usage of the multiple autoimmune syndrome umbrella term proposed by Betterle et al as a replacement for the former APS. This might provide a novel dimension toward a better understanding of the syndrome (12).

Our network analysis and low dimensional visualization using the UMAP algorithm did not confirm the generally applied classification of APS regarding APS II, III, and IV (the analysis of APS I was not possible due to the low number of cases). The current grouping of the syndrome partially overlaps with the cohesive clusters displayed on the UMAPs. This is expected, as both the APS categories and the clusters are determined based on the diseases and their co-occurrence. Our analysis confirmed that HT and GD are corner elements of the network of accumulating autoimmune disorders and showed a special association of HT and T1D as a partially separated subgroup. However, further investigations are required to better define the APS categories. In the current grouping, the emphasis is placed more on the occurrence of a specific disease rather than on its association with either of the 2 corner elements (HT or GD). Furthermore, it is important to consider the clinical relevance of APS III as an all-encompassing category, given the presence of multiple distinct disease combinations in it. Based on our data, the groups of patients APS III collects show a diverse course of syndrome development and organ involvement suggesting that APS III is likely to be overly divergent to form a unified category. Discovering the genetic background might be the most useful information.

Regarding the first manifestations, an interesting finding was that T1D was much more likely to occur as an initial disease than as a later manifestation. So far, this phenomenon has only been examined separately in the APS IV subgroup (10). However, in our study, it was investigated throughout the entire population. Nevertheless, the literature also shows that T1D patients have a significantly higher incidence of additional autoimmunity, which may be explained by the common genetic background and immune regulation of these diseases (27, 33, 34).

The age at the onset of APS in our study was in line with previous literature (35). Furthermore, a remarkable finding was obtained regarding the age at which symptoms first appeared, specifically in relation to sex. This observation adds novel information to the existing body of research. While it was previously understood that females were more affected by autoimmunity, no previous study has shown that males have an earlier onset of APS, particularly in the case of AD if appearing as first manifestation (2, 9, 14-16, 35). The time between diagnosis of the first 2 manifestations varied widely (25, 35, 36). New autoimmune disorders may develop even after 50 years of the initial symptom, which underlines the importance of the follow-up of these patients but makes it challenging to establish a predictive algorithm.

AITD patients were categorized as having HT or GD, so the 2 thyroid diseases did not occur simultaneously in the same patient; however, the sequential appearance of HT and GD may occur in rare cases No such kind of patients were found in our patient group. HT is conventionally characterized by a Th1 pattern of autoimmune reaction, whereas the predominance of Th2 cytokines indicates a humoral type of immune response in GD. Nevertheless, several authors do not separate these 2 entities completely and consider AITDs to be a continuum of conditions ranging from HT at 1 end to GD at the other end of the spectrum (23). Our primary objective in the network analysis was to find differences in the association pattern of other autoimmune diseases between HT and GD. The reason why we have believed that different autoimmune conditions may be associated with HT and GD was that the remission of the autoimmune disorder is common in GD (30-50% of patients remain euthyroid after the cessation of antithyroid medications), while the disappearance of antithyroid antibodies and the resolution of thyroiditis are not found in HT. We found that the similarities between the associated diseases of HT and GD were considerably more apparent than the differences. An almost complete overlap of the associated conditions was found, which raises questions concerning the exact pathophysiological origins of these diseases. However, taking into account their respective associations, the low dimensional mapping also effectively distinguished patients with HT and GD.

CeD was another common disorder observed in the juvenile cohort, in addition to T1D. The prevalence of CeD is rising worldwide (37, 38). There have been many studies recently about the protective effects of gluten-free diet (GFD) in certain autoimmune diseases. Krysiak et al found that the GFD reduced thyroid autoimmunity and slightly increased thyroid output in euthyroid women with HT (39). Another publication reported on the positive effects of GFD in PsO, showing a significant reduction in the psoriasis area and severity index score and also documented resolution of PsO following the adoption of GFD (40). Furthermore, certain studies indicate that GFD may assist in restoring normal metabolic control in individuals with T1D (41).

RA is also thought to be a disease influenced by the microbiome, suggesting that diet and infectious diseases may have an impact on its development. Dysbiosis, periodontitis, and mucosal dysregulation (in the oral, sputum, and vaginal areas) have been observed in patients with RA (42, 43). Interestingly, the most common double combination in the adult cohort was HT + RA followed by HT + T1D and HT + CeD.

A significant discovery from our investigation is that the pattern of secondary manifestations differed substantially from that of T1D, AD, or CeD in cases where thyroid autoimmunity was the initial symptom and that thyroid autoimmunity is frequently associated with gastrointestinal and systemic manifestations. It is also apparent that many of the other most common diseases occur in association with HT or GD. Monitoring the disease progression following the most prevalent first manifestations unveiled the probable subsequent symptoms. HT as the first manifestation was followed by CeD, SS, RA, and AIG, each occurring with a frequency of more than 10%. The most common secondary manifestations in GD were CeD, RA, AD, and CD. Remarkably, over 50% of patients with T1D developed HT, while GD and CeD were also common secondary symptoms, accounting for over 15% of these cases each. Both CeD and AD patients experienced HT in a large number of cases (over 60% of CeD and over 50% of AD cases) with GD being the second most common route. The question at hand is whether there exists a causal biological background behind the co-occurrences, or if they are merely combined based on their frequency. Expanding this study to include a greater number of cases could provide a more comprehensive understanding of the network of disease associations and facilitate the development of a targeted follow-up according to the initial disease. Such a protocol would be highly valuable in terms of both financial and human resources (5, 6, 13, 22, 36, 44). Moreover, understanding the significance of the microbiome's participation in the formation of autoimmune illnesses can aid in developing prevention strategies.

In general, APS is far more prevalent than what is shown in the literature. Implementing screening and follow-up regimens that focus on additional autoimmune comorbidities is crucial for patients with autoimmune disease. Up-to-date registries are essential for accurately assessing the prevalence of the disease.

## Data Availability

All datasets generated during and/or analyzed during the current study are not publicly available but are available from the corresponding author on reasonable request. The code base of all analyses that were conducted in this study can be found on GitHub via the following link: https://github.com/peterkaltenecker/APS_–_A_comprehensive_analysis.

## Abbreviations

AD: Addison’s disease
AIG: autoimmune gastritis
AIH: autoimmune hepatitis
AIHA: autoimmune hemolytic anemia
AITD: autoimmune thyroid disorder
Alo: alopecia
APS: autoimmune polyglandular syndrome
aTg: thyroglobulin antibody
aTPO: thyroid peroxidase antibody
BACH2: BTD Domain and CNC Homolog 2
CD: Crohn's disease
CeD: coeliac disease
CI: confidence interval
CMC: chronic mucocutaneous candidiasis
CTLA4: cytotoxic T- lymphocyte antigen
FCGR2B: Fc fragment of IgG receptor IIb
FCRL3: Fc receptor-like protein 3 gene
GD: Graves’ disease
GFD: gluten-free diet
HLA: human leukocyte antigen
HT: Hashimoto's thyroiditis
hypoPT: hypoparathyroidism
IBD: inflammatory bowel disease
ITP: immune thrombocytopenia
LH: lymphocytic hypophysitis
MAS: multiple autoimmune syndrome
MG: myasthenia gravis
MS: multiple sclerosis
PAPS: primary antiphospholipid syndrome
PASI: psoriasis area and severity index
PBC: primary biliary cholangitis
PM: polymyositis
POF: premature ovarian failure
PSC: primary sclerosing cholangitis
PsO: psoriasis
PTPN22: protein tyrosine phosphatase, non-receptor type 22 gene
RA: rheumatoid arthritis
SLE: systemic lupus erythematosus
SS: Sjögren's syndrome
SSc: systemic sclerosis
T1D: type 1 diabetes mellitus
TSHR: thyroid stimulating hormone receptor antibodies
UC: ulcerative colitis
Vit: vitiligo
VTCN1: V-set domain-containing T-cell activation inhibitor 1
